# Progressive retinal vessel malformation in a premature infant with Sturge-Weber syndrome: a case report and a literature review of ocular manifestations in Sturge-Weber syndrome

**DOI:** 10.1186/s12886-021-01815-8

**Published:** 2021-01-22

**Authors:** Zhengping Hu, Jian Cao, Eun Young Choi, Yun Li

**Affiliations:** 1 Schepens Eye Research Institute, Mass. Eye and Ear, Boston, MA USA; 2 Harvard Medical School, Boston, MA USA; 3 Department of Ophthalmology, The 2nd Xiangya Hospital of Central South University, Changsha, Hunan People’s Republic of China; 4 Hunan Clinical Research Center of Ophthalmic Disease, Changsha, Hunan People’s Republic of China

**Keywords:** Sturge-weber syndrome, Ocular manifestation, Retinal vessel malformation, Nevus flammeus, Diffuse choroidal hemangioma

## Abstract

**Background:**

Sturge-Weber syndrome is a disorder marked by a distinctive facial capillary malformation, neurological abnormalities, and ocular abnormalities such as glaucoma and choroidal hemangioma.

**Case presentation:**

We report a case of progressively formed retinal vessel malformation in a premature male infant with Sturge-Weber syndrome and retinopathy of prematurity, after treatment with intravitreal anti-vascular endothelial growth factor (VEGF). The baby was born at 30 weeks gestation with a nevus flammeus involving his left eyelids and maxillary area. On postmenstrual age week 39, he received intravitreal anti-VEGF. Diffuse choroidal hemangioma became evident at 40 weeks, with the classic “tomato catsup fundus” appearance. These clinical findings characterized Sturge-weber syndrome. He presented with posterior retinal vessel tortuosity and vein-to-vein anastomoses at 44 weeks.

**Conclusion:**

This is a rare case of documented progression of retinal vessel malformations in a patient with Sturge-Weber syndrome and retinopathy of prematurity.

## Background

Sturge-Weber syndrome (SWS) is a rare congenital phakomatosis defined by several vascular anomalies, including facial capillary malformations (nevus flammeus) and ipsilateral leptomeningeal angiomatosis, as well as ocular abnormalities which can involve the eyelid, bulbar conjunctiva, cornea, anterior chamber, choroid, and retina, mostly ipsilateral to the nevus flammeus [1, 2]. Infants with hemifacial and forehead nevus flammeus phenotypes are at the highest risk of SWS (45–80%) [3]. The estimated incidence of SWS is 1:50,000 live births, with no significant difference between males and females [4]. A somatic mutation in the GNAQ gene has been reported to be associated with the pathogenesis of SWS [5].

The spectrum of SWS is classified as follows: Type I is the most common presentation that involves both facial and leptomeningeal angiomas and glaucoma may be present. Type II involves facial angioma (without the involvement of the central nervous system) and may include glaucoma. Type III is characterized by leptomeningeal angiomas only, with no facial involvement, and glaucoma is rarely seen. The case presented in this report was classified as SWS Type II [6]..

## Case presentation

**Fig. 1:**
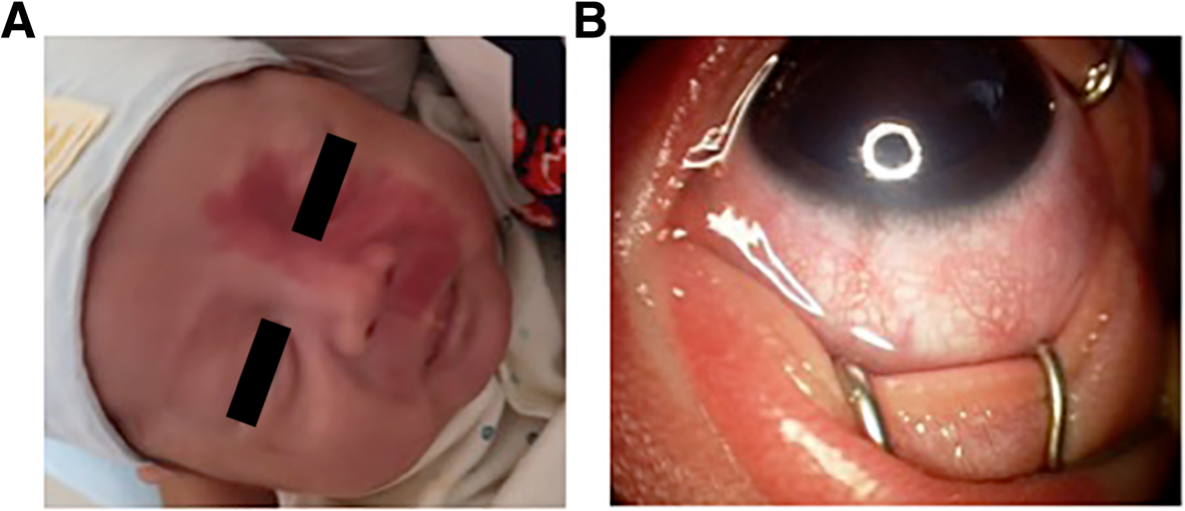
Vascular abnormalities of the face and anterior segment in a patient with Sturge Weber syndrome. **a** Nevus flammeus on the left side of the face. **b** Retcam image, showing increased epibulbar and conjunctival vasculature in the left eye

**Fig. 2:**
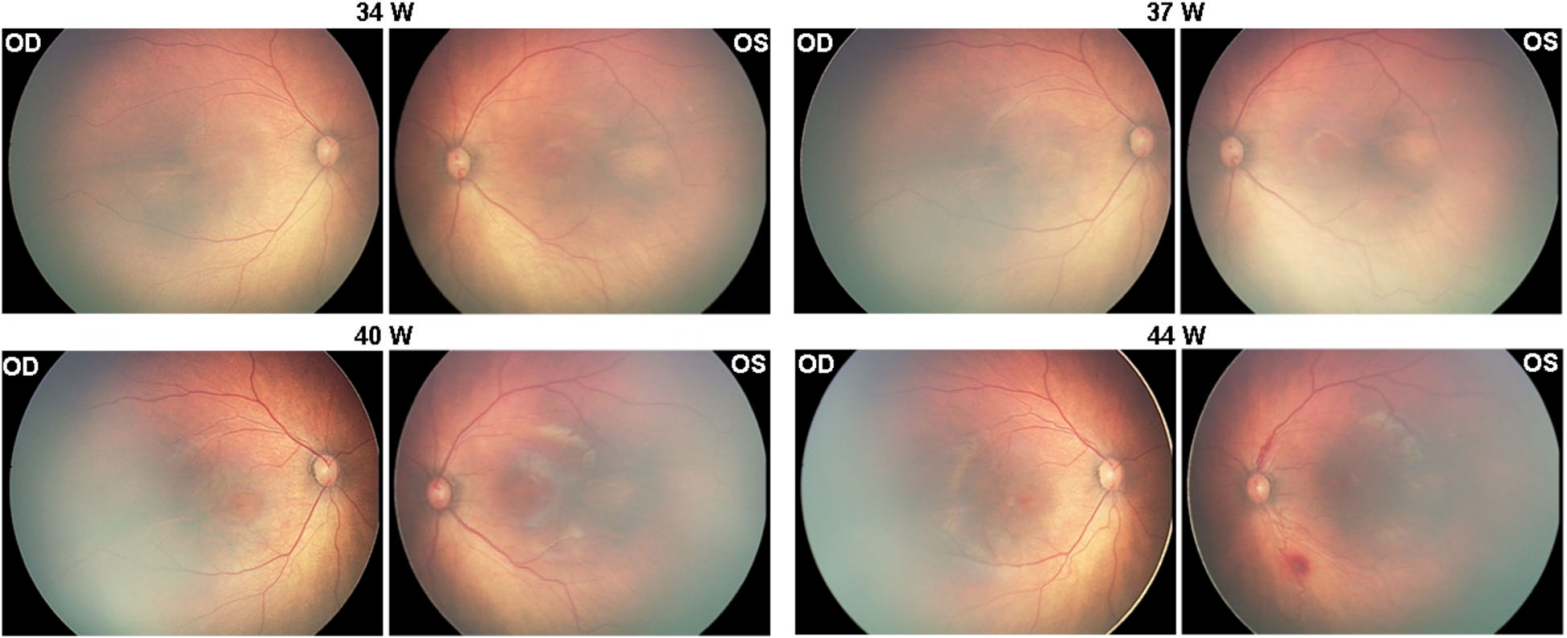
Progression of the “tomato catsup fundus”. Retcam funduscopy images showing progression of the”tomato-catsup fundus” in a patient with Sturge-Weber syndrome and ROP. The images show the gradual formation of a bright reddish color of the fundus (tomato catsup fundus) in the affected left eye compared to the healthy right eye from PMA 34 week to 40 week, which is the characteristic sign of DCH

**Fig. 3:**
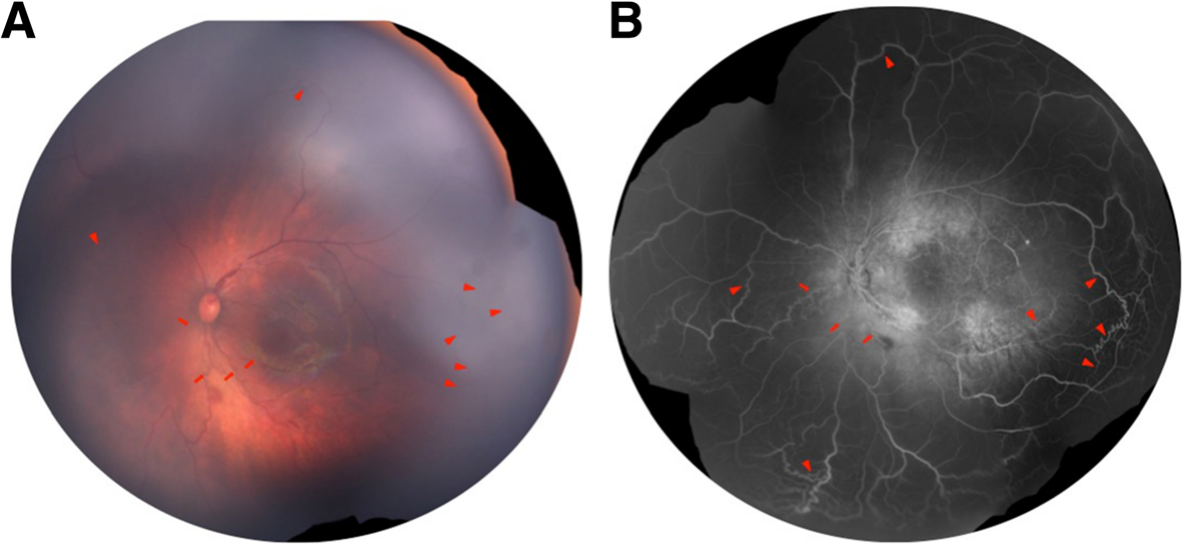
Fundus photograph and fundus fluorescein angiography (FFA) of the left eye **a** Fundua photograph and **b** FFA showed striking tortuosity of posterior retinal vessels (arrows) and multiple vein-to-vein anastomoses (arrowheads) at the periphery, which is better appreciated in FFA. Marked hyper-fluorescent lesions at the posterior pole on recirculation phase of FFA correspond to DCH. The right eye was normal on both fundoscopy and FFA

A premature male infant (gestational age 30 weeks, birth weight 1410 g) was screened for retinopathy of prematurity (ROP). The baby was naturally delivered, with a short time of oxygen inhalation after birth. On examination, at age postmenstrual age (PMA) 34 weeks (initial visit) a nevus flammeus was noted on his left eyelids and maxillary area, following the V1 and V2 distributions of the trigeminal nerve (Fig. 1a). From PMA 37 weeks, he was found to have progressive ROP (Zone 2 stage 3) in his left eye and received intravitreal ranibizumab (Lucentis, Novartis) at PMA 39 weeks to treat type 1 ROP. Informed consent was obtained from the parents to use ranibizumab to treat the ROP. The ridge and neovascularization regressed satisfactorily, but diffuse choroidal hemangioma (DCH) became evident at 40 weeks, with the classic “tomato catsup fundus” appearance. These clinical findings characterized Sturge-Weber syndrome. At PMA 44 weeks, the baby was noted to have increased bulbar conjunctival vascularization and retinal vascular tortuosity. Retcam images showed vein-to- vein anastomoses in the peripheral retina (Fig. 2). Retinal vascular tortuosity and vein-to-vein anastomoses were better appreciated on fundus fluorescein angiography (FFA) under general anesthesia (Fig. 3). The patient continues to have a normal IOP and is under close observation for further ocular changes. Retcam revealed conjunctiva vasodilation in the left eye; no other changes were identified in the anterior chamber. The right eye was normal upon examination. Magnetic resonance imaging did not show leptomeningeal angiomatosis (result not shown).

## Discussion and conclusions

Ocular alterations in SWS are seen in approximately 30–60% of patients [7]. The vascular anomalies include the facial nevus flammeus [1, 2], ipsilateral leptomeningeal angiomatosis [8], diffuse choroidal hemangioma [9–29], and rarely retinal vascular anomalies [30, 31]. The GNAQ gene, involved in regulating intracellular signaling pathways [32], has been identified as a potential cause for SWS [5]. Bichsel et al. reported recently that SWS patients have a high GNAQ R183Q mutant allelic frequency, which was comparable to brain tissue from a different set of patients with SWS. Three common somatic mutations of GNAQ were not detected in the patient’s choroidal hemangioma tissue in their study [33]. On the other hand, Huang reported that somatic GNAQ p.R183Q mutation is enriched in endothelial cells in SWS brain lesions [34]. Nakashima further confirmed the somatic GNAQ mutation c.548G>A (p.R183Q) in SWS patients [35].

SWS can be found coexisting with other rare phakomatoses or vessel malformations like Klippel-Trenaunay syndrome [36], Wyburn Mason Syndrome [37], and Moyamoya disease [38].

To summarize the ocular manifestations in SWS, we performed a literature search of papers dated from Jan. 1, 1990 to Feb. 28, 2019, using “Sturge–Weber syndrome”, “Sturge-Weber and eye”, “Sturge-Weber and ocular”, “Sturge-Weber and retinal” and “Sturge-weber and glaucoma” as keywords in the PubMed database. The content of each paper was thoroughly checked to ensure its relevance to the topic. Cases in English were checked by the full text. Papers in non-English related to SWS were checked by their English title and abstract. Cases concerning SWS ocular complications were recorded and analyzed.

PubMed search yielded 123 cases diagnosed with “Sturge-Weber syndrome”. Among these, 89 (69.6%) cases with ocular involvement were included. Cases of SWS with ocular trauma and SWS in association with other diseases that may have ocular involvement were excluded. Of the 89 cases, 40 (44.94%) were female and 49 (55.05%) were male. The patients’ ages ranged from 2 weeks to 66 years old (mean 20.65 years). Ocular complications observed in SWS are diverse and summarized in Table 1**.** Cataract and other changes secondary to glaucoma, only glaucoma was recorded.

**Table 1:** Ocular complications in Sturge-Weber syndrome

Complications	Case (percentage of total)	Reference
Glaucoma	36 (40.45%)	[9, 10, 30, 31, 39–68]
Diffuse Choroidal Hemangioma	39 (43.82%)	[10–29, 31, 47, 55, 57–60, 62, 64, 69]
Circumscribed choroidal hemangioma	4	[70–73]
Retinal vascular anomaly	5	[10, 11, 41, 74, 75]
Intraocular osseous metaplasia	1	[39]
Ocular Melanocytosis	1	[11]
Iris Mammillations, neovascularization, atrophy	3	[11, 68, 73]
Increased Choroidal Thickness	22 (24.72%)	[20, 76, 77]
Episcleral hemangioma, vessel malformation	6	[54, 65, 67, 73, 78, 79]
Retinal detachment	1	[80]
Eyelid hemangioma	1	[29]
Central retinal vein occlusion	1	[61]
Ectopia lentis	1	[81]
Optic neuropathy	1	[82]

DCH was the most common ocular complication associated with SWS, seen in 40.45% of patients in our literature review [3–11, 30–68]. In addition to the typical characteristics of DCH, an early sign could be unilateral anisometropia due to DCH [55]. There were 4 cases of circumscribed choroidal hemangioma in SWS patients. Althaus et al. [73] documented a contralateral circumscribed choroidal hemangioma; the patient had a left-sided naevus flammeus and a right-sided circumscribed choroidal hemangioma. Complications of DCH are macular edema, exudative retinal detachment, pigmentary changes within the RPE, subretinal fibrosis, and orange pigment changes secondary to diffuse choroidal hemangioma [10]. DCH can also be associated with increased choroidal thickness in the fellow eye with no visible hamangioma [76, 77]. Spectral domain optical coherence tomography (SD-OCT) has shown that choroidal thickness of the affected eyes, ipsilateral to facial naevus flammeus, is twice that of the fellow eyes [10]. Chavala et al. reported a patient with a bilateral facial nevus flammeus who developed exudative retinal detachments in both eyes without mentioning the diffuse choroidal hemaniogma [80]. A case of unique subretinal osseous metaplasia in SWS with endophthalmitis, glaucoma and complicated cataract was reported by Pavlenko et al [39]

Glaucoma is another frequent ophthalmic complication of SWS, occurring in 43.82% of all cases [9, 30, 31, 38–68]. It can be further classified into early-onset glaucoma and late-onset juvenile glaucoma. The proposed etiologies of glaucoma in SWS include buphthalmia, anterior chamber angle mal-development, and raised episcleral venous pressure [3, 83]. Acute primary angle-closure glaucoma, which is rare in SWS patients, has been reported by Su [9] and Lambiase et al [40] Other commonly observed anterior segment abnormalities include conjunctival, episcleral, and iris vessel dilation and hemangioma. Corneal changes related to congenital glaucoma can also be seen. Secondary cataract has been reported in many cases; Moore et al. [81] report a case of ectopia lentis associated with angle-closure glaucoma without historical trauma. Iris mammilliations are usually observed when SWS is associated with melanocytosis in the context of phakomatosis pigmentovascularis that can further increase the risk of glaucoma [84].

Retinal vascular anomaly is rare in SWS, but when observed, it is often associated with choroidal hemangiomas. Previously documented anomalies include retinal arteriovenous communications [75], a white appearance of retinal vessels, reduced perfusion of the venous system [74], vein-to-vein anastomoses, and retinal venous dilatation and tortuosity [11, 41]. Two of the reported cases were bilateral [63]. Venous congestion of the left upper venous branch was observed in the left eye of a case with bilateral DCH [10].

We describe a premature infant, the youngest SWS patient ever reported, who presented with a left-sided facial nevus flammeus at birth and progression of multiple ocular vessel malformations. The infant had a normal fundus color at the initial visit. We recorded the development of DCH in the affected eye. The color of the fundus turned orange-red, compared to the healthy eye as we showed in the figure, and gradually revealed the characteristic “tomato catsup” fundus. The progression of retinal vessel tortuosity associated with multiple vein-to-vein anastomoses present in SWS patients has rarely been reported. The patient was also diagnosed with progressive ROP in his left eye at PMA 37. ROP is an ocular disease characterized by the onset of vascular abnormalities in the developing retina, in which the premature and incompletely vascularized retinas may be obliterated by stressors, namely, oxygen supply. The abnormal vessels in ROP are usually anarchic, leaky, and excessive, which results in the invasion of the vitreous and involves retina traction and bleeding [85]. From PMA 40 weeks and after, we observed the DCH in the left eye, with retinal vessel tortuosity and vein-to-vein anastomoses, which is not similar to retinal vessel abnormality usually seen in ROP. Furthermore, anti-VEGF has been shown effective in halting ROP progression [86]. In the case reported herein retina traction and neovascularization in ROP regressed satisfactorily, but the retinal vessel tortuosity did not regress.

In conclusion we report a gradual formation of DCH and retinal vessel malformation in an infant. This suggests that babies born with nevus flammeus should be checked repeatedly instead of one-time screening, especially in premature babies.

## Data Availability

Data and materials are available upon request from the corresponding author at yun.li@csu.edu.cn.

## Abbreviations

SWS: Sturge-Weber syndrome
RPE: Retinal Pigment Epithelial
FFA: Fundus Fluorescein Angiography
IOP: Intraocular Pressure
MRI: Magnetic Resonance Imaging
ROP: Retinopathy of Prematurity
VEGF: Vascular Endothelial Growth Factor
DCH: Diffuse Choroidal Hemangioma
CNV: Choroidal Neovascularization
PMA: Post Menstrual Age
OCT: Optical Coherence Tomography
SD-OCT: Spectral-domain Optical Coherence Tomography
